# A quantitative analysis of extension and distribution of lung injury in COVID-19: a prospective study based on chest computed tomography

**DOI:** 10.1186/s13054-021-03685-4

**Published:** 2021-08-04

**Authors:** Mariangela Pellegrini, Aleksandra Larina, Evangelos Mourtos, Robert Frithiof, Miklos Lipcsey, Michael Hultström, Monica Segelsjö, Tomas Hansen, Gaetano Perchiazzi

**Affiliations:** 1 Anesthesiology and Intensive Care, Department of Surgical Sciences, Uppsala University, Akademiska sjukhuset, Uppsala, Sweden; 2 Radiology, Department of Surgical Sciences, Uppsala University, Uppsala, Sweden; 3 Hedenstierna Laboratory, Department of Surgical Sciences, Uppsala University, Uppsala, Sweden; 4 Integrative Physiology, Department of Medical Cell Biology, Uppsala University, Uppsala, Sweden

**Keywords:** COVID-19, SARS-CoV2, ARDS, Acute respiratory distress syndrome, Mechanical ventilation, Computed tomography

## Abstract

**Background:**

Typical features differentiate COVID-19-associated lung injury from acute respiratory distress syndrome. The clinical role of chest computed tomography (CT) in describing the progression of COVID-19-associated lung injury remains to be clarified. We investigated in COVID-19 patients the regional distribution of lung injury and the influence of clinical and laboratory features on its progression.

**Methods:**

This was a prospective study. For each CT, twenty images, evenly spaced along the cranio-caudal axis, were selected. For regional analysis, each CT image was divided into three concentric subpleural regions of interest and four quadrants. Hyper-, normally, hypo- and non-inflated lung compartments were defined. Nonparametric tests were used for hypothesis testing (*α* = 0.05). Spearman correlation test was used to detect correlations between lung compartments and clinical features.

**Results:**

Twenty-three out of 111 recruited patients were eligible for further analysis. Five hundred-sixty CT images were analyzed. Lung injury, composed by hypo- and non-inflated areas, was significantly more represented in subpleural than in core lung regions. A secondary, centripetal spread of lung injury was associated with exposure to mechanical ventilation (*p* < 0.04), longer spontaneous breathing (more than 14 days, *p* < 0.05) and non-protective tidal volume (*p* < 0.04). Positive fluid balance (*p* < 0.01), high plasma D-dimers (*p* < 0.01) and ferritin (*p* < 0.04) were associated with increased lung injury.

**Conclusions:**

In a cohort of COVID-19 patients with severe respiratory failure, a predominant subpleural distribution of lung injury is observed. Prolonged spontaneous breathing and high tidal volumes, both causes of patient self-induced lung injury, are associated to an extensive involvement of more central regions. Positive fluid balance, inflammation and thrombosis are associated with lung injury.

*Trial registration* Study registered a priori the 20th of March, 2020. Clinical Trials ID NCT04316884.

**Supplementary Information:**

The online version contains supplementary material available at 10.1186/s13054-021-03685-4.

## Introduction

The pandemic caused by the novel Severe Acute Respiratory Syndrome Coronavirus-2 (SARS-CoV2) and named Coronavirus Disease 2019 (COVID-19) has affected millions of people and caused thousands of deaths worldwide [1]. Although most of the times characterized by a favorable prognosis, when particularly severe COVID-19 is associated with acute respiratory failure, significant hypoxemia and high mortality rate [2]. SARS-CoV2 has been classified among those causes potentially leading to acute respiratory distress syndrome (ARDS) [3, 4]. In previous observations [5], a substantially high compliance was described in mechanically ventilated patients affected by COVID-19 ARDS, emphasizing the atypical nature of COVID-19 ARDS compared to classical ARDS. This difference has not been confirmed by more recent studies [6]. To which extent COVID-19 ARDS differs from a classical ARDS and whether this difference matters from a diagnostic and therapeutic point of view is still an unsolved and highly debated issue [7–9]. The mechanisms leading to the evolution of lung injury toward the most severe forms of acute respiratory failure in COVID-19 still need better clarification. The asymptomatic hypoxemic hyperpnoea and high respiratory drive leading to patient self-induced lung injury (P-SILI) [10, 11], as well as the activation of a cytokine storm [12], have been proposed as potential causes leading the progress of lung injury in COVID-19 patients. To date, no data fully support these hypotheses [13, 14].

Chest computed tomography (CT) is the reference imaging technique during ARDS [15]; however, the current clinical practice for COVID-19 patients considers CT as a complementary, not strictly needed examination [16]. Several studies have reported an apparent subpleural distribution of lung injury in COVID-19, but the distribution has not been objectively quantified, nor has the progression of injury been measured [13, 17–20].

We analyzed chest CT scans from prospectively included COVID-19 patients in need of intensive care. We examined morphological, clinical and functional features in order to investigate their possible correlations. Specifically, we investigated in COVID-19 patients the regional distribution of lung injury and the influence of clinical and laboratory features on its progressive extension. We tested:

1. The hypothesis that lung injury has mainly a subpleural distribution coexisting with a varying involvement of the core areas of the lung;
2. The correlation between the core distribution of lung injury, measured by numerical analysis of CT images, and clinical features characterizing critically ill patients (days and pattern of mechanical ventilation, positive fluid balance, markers of inflammation and thrombotic state).

## Methods

This study was performed in patients admitted to the intensive care units (ICU) of Uppsala University Hospital (Sweden). The presented data are part of a study approved by the National Ethical Review Agency (EPM; No. 2020-01623). The Declaration of Helsinki and its subsequent revisions were followed. The protocol was registered a priori (Clinical Trials ID: NCT04316884). STROBE guidelines were followed for reporting. Informed consent was obtained from the patient or from next of kin if the patient was unable to give consent. Selected patients were older than 18 years, positive PCR test for SARS-CoV2 on nasal swab specimen, admitted to intensive care unit between March 13 and June 5, 2020 and undergone a spiral chest CT. The CT scans were performed without contrast agent, covering the whole lung parenchyma in supine position. Spiral CT scans were performed when clinically indicated and feasible, hence at different stages of the disease. Comprehensive clinical data were collected on daily basis (Tables 1, 2).

**Table 1:** Descriptive statistics of all COVID-19 patients included in the study

	All patients		Patients without CT		Patients with CT		
Numerosity	111		88		23		
Sex (males, %)	82	74	67	78	15	68	

	Median	IQR	Median	IQR	Median	IQR	*p*
Age	61	21	57	20	68	13	0.02*
BMI	29	7	29	6	27	6	0.21
Length of stay in ICU (days)	9	10	7	9	13	15	0.02*

Admission physiology	Median	IQR	Median	IQR	Median	IQR	*p*
Respiratory rate (breaths/min)	28	13	28	14	28	15	0.61
Heart rate (beats/min)	89	22	88	20	92	25	0.44
Mean arterial pressure (mmHg)	89	18	90	20	86	13	0.31
Body temperature (°C)	38	1	38	1	37.5	0.5	0.86
SAPS3	52	10	51	10	57	13	< 0.01*

Mortality	N	%	N	%	N	%
Dead at 30 days	22	20	14	16	8	36

Premorbidities	N	%	N	%	N	%
Lung disease	28	26	24	28	3	16
Pulmonary hypertension	0	0	0	0	0	0
Smokers (previous or ongoing)	12	24	25	26	8	21
Hypertension	56	52	42	49	12	63
Heart failure	5	5	4	5	1	5
Ischemic heart disease	11	10	7	8	4	21
Vascular disease	19	18	13	15	6	32
Thromboembolic event	10	9	7	8	2	11
Liver failure	1	1	1	1	0	0
Malignant disease	7	6	3	3	3	16
Diabetes	29	27	20	23	7	37
Neurological disease	6	6	6	7	0	0
Psychiatric disease	12	11	11	13	1	5

Preadmission treatment	N	%	N	%	N	%
Steroid treatment	12	11	8	9	3	16
ACEi or ARB	40	37	30	35	9	47
Anticoagulation treatment	24	22	16	19	7	37

Data were reported as median values (IQR) or numerosity (%). Wilcoxon rank sum test to detect statistical differences between patients underwent and patients who did not undergo chest CT (*a* = 0.05, * to mark statistical significant differences)

**Table 2:** Descriptive statistics of COVID-19 patients underwent chest CT

Patients underwent chest CT	Numerosity = 23			
	At arrival		The day of CT	
ARDS	N	%	N	%
Mild	3	11	2	7
Moderate	13	46	23	93
Severe	12	43	0	0

	At arrival		The day of CT		*p*
	Median	IQR	Median	IQR	
Days from symptoms onset	11	3	18	14	< 0.01*
Weight [kg]	80	23	90.5	29	< 0.01*
Days of dialysis			0	6	
Cumulative fluid balance [mL]			4728	4043	
Prone position [hours]			1	30	
Muscle Relaxation [hours]			19	15	
Spontaneous breathing [days]			15	12	

	Total		The day of CT		*p*
	Median	IQR	Median	IQR	
Days of invasive mechanical ventilation	7	16	4	11	0.15

Laboratory analysis	At arrival		The day of CT		*p*
	Median	IQR	Median	IQR	
C-reactive protein [mg/mL]	200	131	202	209	0.69
Leukocytes [10^3^/mL]	9	3	11	10	0.09
Procalcitonin [ng/mL]	1.10	1.44	1.00	2.30	0.75
Interleukin-6 (IL-6) [pg/mL]	166	167	107	122	0.33
Ferritin [µg/L]	1457	1685	1094	1984	< 0.01*
D-dimer [ng/mL]	1.95	1.63	2.45	3.83	< 0.01*
Creatinine [umol/mL]	89	44	102	133	< 0.01*
Glomerular filtration rate (creatinine) [mL/min/1.73 m^2^]	64	30	57	57	< 0.01*
P/F ratio [mmHg]	104	75	164	50	< 0.01*
PCO_2_ [mmHg]	33	9	42	12	< 0.01*
pH	7.48	0.05	7.43	0.15	0.01*
Respiratory rate (breath per minutes)	31	13	23	7	< 0.01*

Respiratory mechanics	The day of CT	
	Median	IQR
Highest Vt/kg PBW	6.8	0.9
Highest minute ventilation [L/min]	11.7	3.6
PEEP [cmH_2_O]	12	4
Lowest static compliance [mL/cmH_2_O]	38	25
Highest plateau pressure [cmH_2_O]	14	7
Highest peak pressure [cmH_2_O]	22	6

		Median	IQR
Days of noninvasive ventilation	(5 out 23 patients)	1.5	2

Data were reported as median values (IQR) or numerosity (%). Wilcoxon rank sum test to detect statistical differences (a = 0.05, * to mark statistical significant differences)

### CT image analysis

**Fig. 1:**
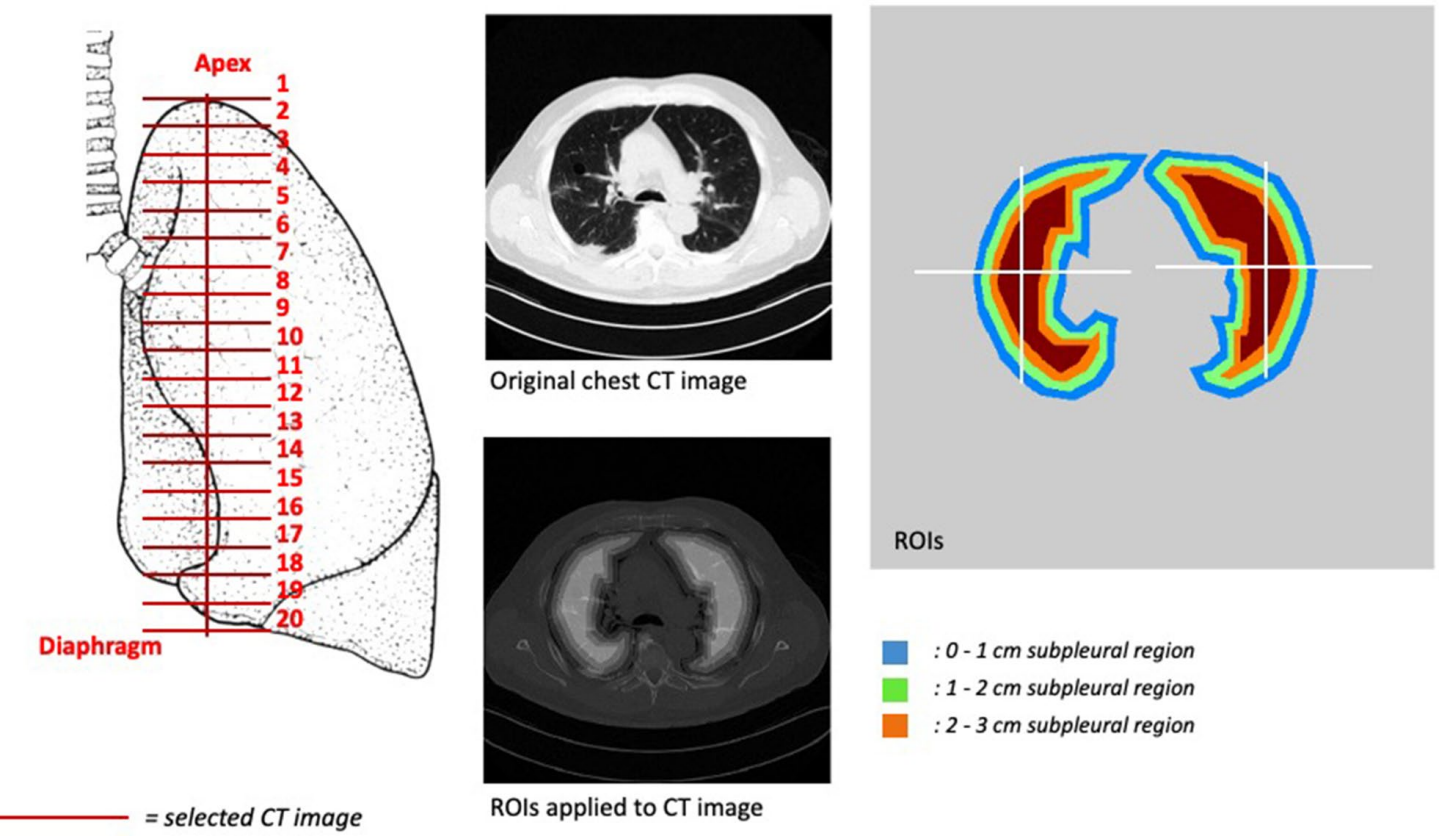
CT image analysis. Left panel. Coronal representation of lungs. Twenty CT images were selected along the cranial-caudal axis. Central panel. Upper part: representative image of an original CT image from a patients included in the study. Lower part: region of interest (ROI) applied to CT image. Right panel: representative image of three ROIs (areas concentric to the visceral pleura: between 0 and 1 cm in blue; 1 and 2 cm in green; and 2 and 3 cm in orange) and quadrants: internal dependent, external dependent, internal non-dependent + external non-dependent). The identification of quadrants was based on the detection of the centroid (or geometrical center) univocally characterizing each lung for each analyzed CT image

For each chest CT scan, twenty images, evenly spaced along the cranial-caudal axis between the apex and the diaphragmatic dome, were selected for analysis [21, 22]. In order to describe the progress of lung lesions along the subpleural-to-core direction inside lung parenchyma, three concentric subpleural regions of interest were defined: from pleural surface to 1 cm depth; from 1 to 2 cm depth; and from 2 to 3 cm depth. To distinguish between hypodensities related to lung injury and hypodensities due to dependent atelectasis and pleural effusion, each lung was divided into four quadrants. Consequently, three functional regions were identified: *non-dependent* (external non-dependent + internal non-dependent), *dependent* (external dependent + internal dependent) and *external* (external non-dependent + external dependent). Concentric ROIs and lung regions were therefore combined for regional analysis (Fig. 1).

**Fig. 2:**
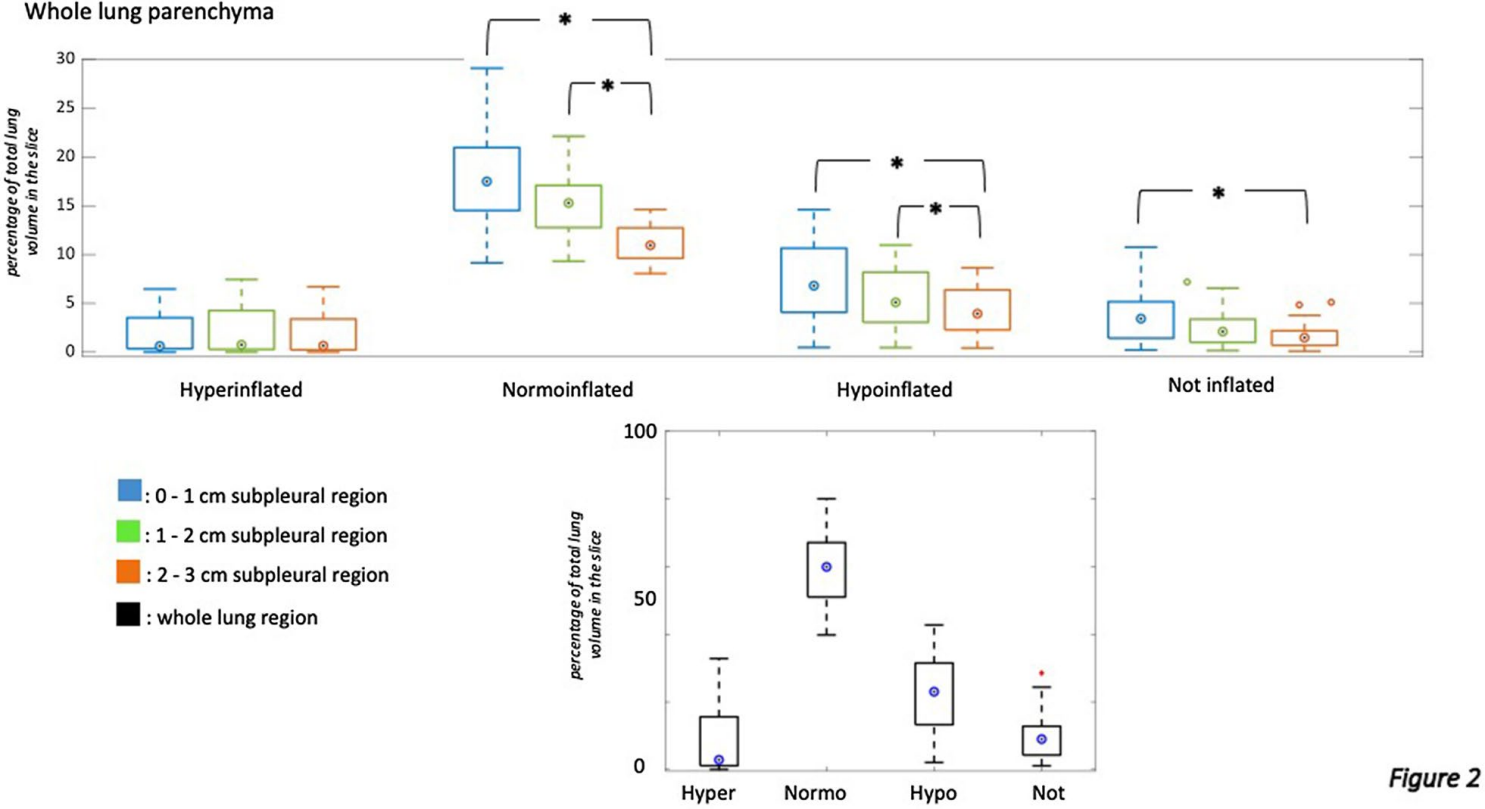
Boxplot showing regional distribution of lung compartments in the whole lung parenchyma. Each subgroup of boxplot shows how lung compartments are divided among different subpleural regions of interest (blue for 0–1 cm subpleural area, green for 1–2 cm subpleural area, orange for 2–3 cm subpleural area, black for the whole analyzed area). The regional distribution of lung compartments is expressed in % of total lung volume for each CT slice (y-axis). Friedman's test was used to detect statistical differences. Pairwise comparisons (adjustment for multiple comparisons was applied according to the Bonferroni method) were used if the analysis of variance detected a significant difference inside the tested group of ROIs. * to indicate statistical differences

**Fig. 3:**
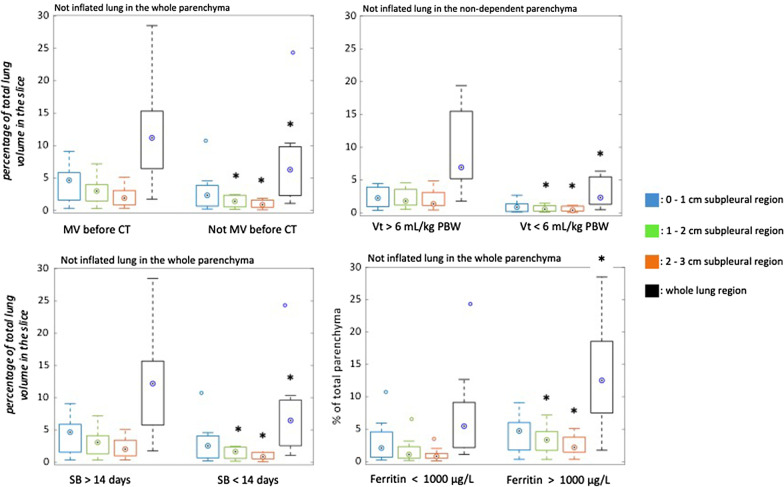
Regional distribution of non-inflated lung compartment in patients’ subgroups defined based on dichotomous variables. **A** exposure to mechanical ventilation before the chest CT (yes/not); **B** exposure to a mean tidal volume higher/lower than 6 mL/kg PBW; **C** duration of spontaneous breathing for more/less than 14 days before the chest CT; **D** plasma ferritin lower/higher than 1000 ug/L. * to indicate statistical difference. The regional distribution of lung compartments is expressed in % of total parenchyma (y-axis) for all the reported graphs. For p-values, see Supplementary materials. Vt: tidal volume, PBW: predicted body weight, SB: spontaneous breathing

Each voxel composing the CT images was characterized by a CT number expressed as Hounsfield units (HU). Following a previous convention [23], four lung compartments were defined: hyperinflated (HU between − 1000 and − 800), normoinflated (HU between − 800 and − 500), hypoinflated (HU between − 500 and − 100) and non-inflated or atelectasis (HU between − 100 and + 100). The extension of each lung compartment was expressed as percentage of the total lung volume analyzed in a given chest CT slice (defined in Figs. 2 and 3 as *% of total lung volume*). The extension of pathologically altered lung was defined as the sum of hypoinflated and non-inflated (atelectatic) parenchyma [24, 25] distributed in not-dependent or external regions. For each of the 20 slices selected per CT scan, lung volume, gas volume and lung weight have been calculated according to established methods [21, 22, 26] applying the following equations:

$${\text{Volume}}\,{\text{of}}\,{\text{gas}} = {\text{voxel}}\,{\text{volume}}*{\text{voxel}}\,{\text{attenuation}}/ - 1000$$

$${\text{Tissue}}\,{\text{weight}} = {\text{voxel}}\,{\text{volume}}*\left( {1 \, - \, \left( {{\text{voxel}}\,{\text{attenuation}}/ - 1000} \right)} \right)$$

When the voxel volume is expressed as cubic centimeters [cm^3^], the volume of gas is in milliliters [ml] and the tissue weight is in grams [g]; voxel attenuation is expressed as Hounsfield units [HU].

Applying the above-mentioned equations, total lung volume, total gas volume and total lung weight have been then calculated from the sequence of CT scans, following the interpolation method reported by Ball et al. [22] and by Reske et al. [21]:

$$Mlung= \sum_{i=1}^{N-1}\left(f* \frac{{M}_{i}+{M}_{i+1}}{2*t}\right)+ \frac{{M}_{1}+{M}_{N}}{2}$$

where *N* is the number of slices, *t* is the slice thickness, *f* the distance between slices, and *M*_*i*_ the lung mass in the ith slice. This equation yields the total mass of the lung *Mlung*. This general interpolation method is also able to compute total lung volume and total gas volume, when the mass *Mi* is appropriately substituted by, respectively, the lung volume or the gas volume in the *i*th slice.

The mentioned equation was used for computing lung volume, gas volume and lung weight for the entire lung as well as for each lung aeration compartment. The latter have been reported both as absolute values and as percentage of the total lung parenchyma.

Patients’ subgroups were defined based on dichotomous variables: 1) exposure to mechanical ventilation before CT scan (yes/not); 2) exposure to a mean tidal volume higher/lower than 6 mL/kg predicted body weight (PBW); 3) duration of spontaneous breathing for more/less than 14 days from the onset of symptoms; 4) plasma ferritin lower/higher than 1000 µg/L (normal plasma ferritin range: 12 to 150 µg/L). Spontaneous breathing, before invasive ventilation, was defined as all types of assisted and not-assisted noninvasive ventilation. The selected thresholds for spontaneous breathing duration and for plasma ferritin were based on previous literature [27].

### Statistical analysis

Continuous variables were reported as medians and interquartile ranges (IQR). Friedman's test was used to detect statistical differences in HU distribution among the three subpleural layers and the whole lung parenchyma. Pairwise comparisons were made when analysis of variance detected a significant difference inside the tested groups. The adjustment for multiple comparisons was applied by using the Bonferroni method. A Wilcoxon rank sum nonparametric test was used to evaluate statistical differences between subgroups defined by dichotomous variables. Spearman correlation test was used to analyze correlations between lung compartments and clinical data. Pairwise linear correlation coefficients (*R*_s_) and p-values were collected and interpreted based on *α* = 0.05.

More detailed Materials and methods are reported as Additional file 1.

## Results

One hundred-eleven patients affected by severe COVID-19 and requiring intensive care were prospectively recruited in the study. Twenty-three out of 111 patients underwent at least one chest CT and hence were eligible for the current analysis. Five out of 23 underwent two chest CT scans, and a total of 560 CT images were analyzed. Overall, 28 chest CTs were independently included in the analysis. In the group of patients undergoing the CT scan, the median age was 68 (IQR 13) years, and 15 (68%) were men. Median ratio of partial pressure of arterial oxygen to fractional concentration of oxygen in inspired air the day of the CT was 164 mmHg (IQR 50) and SAPS3 score at ICU admission was 57 (IQR 13). Eight (36%) out of 23 patients died by day 30 after ICU admission (Tables 1, 2). The analysis of total lung volume, gas volume and lung weight is reported in Table 3.

**Table 3:** Lung volume, gas volume and lung weight calculated for the entire lung parenchyma as well as for each lung aeration compartment (hyper-, normally, poorly and non-inflated)

CT variable	Lung volume (ml)		Lung volume (%)		Gas volume (ml)		Gas volume (%)		Weight (g)		Weight (%)	
	Median	IQR	Median	IQR	Median	IQR	Median	IQR	Median	IQR	Median	IQR
Total lung	2103	1240			1154	1095			919	411		
Hyperinflated	500	748	23	30	391	664	35	25	83	89	9	12
Normally inflated	1000	539	47	12	594	380	51	16	285	166	31	18
Poorly inflated	400	246	19	11	143	76	12	13	312	170	34	11
Non-inflated	200	280	11	13	26	12	2	2	239	254	26	18

The latter have been reported as absolute values as well as percentage of the total lung parenchyma. Data are reported as median values and interquartile ranges (IQR)

### Regional distribution of lung hypodensities

The distribution of lung compartments among the analyzed lung regions is reported in Fig. 2 and Additional file 2: Figures S1–S2. Hyperinflated parenchyma was more represented in non-dependent regions and equally distributed at different distances from the pleura. The dependent lung regions were characterized by more extended hypodensities (hypo- and non-inflated lung) compared to non-dependent lung regions. This at the expenses of normoinflated lung regions (Additional file 2: Figure S2 and Tables S1–S2). Normoinflated, hypoinflated and non-inflated compartments were more common in lung regions close to the pleura (0 to 1 cm below pleura) compared to more central ones (2 to 3 cm). This was true independently from the selected quadrant and was found in not dependent as well as in dependent lung regions, hence, showing that the distribution of hypo- and non-inflated compartments was not only influenced by gravitational forces (Fig. 2, Additional file 2: Figures S1–S2 and Tables S1–S2).

### Correlations between lung compartments and clinical features

#### Dichotomous variables

The extension of non-inflated injured lung visualized by CT was affected by the exposure to mechanical ventilation (19 out of 28) as well as by the duration of spontaneous breathing before the chest CT scan was performed (Fig. 3 and Additional file 2: Table S3). Patients who breathed spontaneously for a period longer than 14 days before the CT scan (18 out of 28) showed a significantly larger extension of non-inflated parenchyma compared to those patients spontaneously breathing for less than 14 days (10 out of 28) (Fig. 3). Moreover, for those patients exposed to invasive (controlled and assisted) mechanical ventilation, a tidal volume higher than 6 mL/kg PBW (8 out of 19) was one of the factors associated with a more extended hypodensities related to lung injury visualized by CT (Fig. 3 and Additional file 2: Table S3). A ferritin level higher than 1000 µg/L (11 out of 28), sampled the day of the CT scan, was associated with a broader non-inflated injured lung parenchyma (Fig. 3 and Additional file 2: Table S3).

### Continuous variables

The statistically significant correlations are presented in Additional file 2: Tables S4 and S5. A direct correlation (positive R_s_) was found between the amount of non-inflated lung and respiratory rate at ICU admission. An inverse correlation (negative R_s_) was found between the amount of hypoinflated lung and pH at ICU admission. The hypoinflated lung correlated with days of symptoms, duration of mechanical ventilation, renal replacement therapy (RRT), prone positioning and muscle relaxation. Measured tidal volume (mL/kg PBW) was correlated with the extension of hypoinflated lung in patients on mechanical ventilation. A positive cumulative fluid balance and plasma D-dimer were correlated with the extent of injured lung. Moreover, injured lung was inversely correlated with peripheral oxygen saturation, and normally ventilated lung was inversely correlated with partial pressure of carbon dioxide.

## Discussion

A numerical analysis was applied to CT scans to quantify morphology and extension of lung alterations in COVID-19. In the studied population, a relatively high portion of lung volume (30%) was composed by poorly and non-aerated parenchyma, characterized by low gas volume (169 out of 1154 ml) and high lung weight (551 out of 919 g). See Table 3.

The most important finding of the current study was that lung injury characterizing COVID-19 mainly affected subpleural lung regions (Fig. 2 and Additional file 2: Figure S1). Moreover, lung injury involved more central regions in patients who underwent potentially dangerous ventilation (Fig. 3). In particular, spontaneous breathing for longer than 14 days from the onset of symptoms, a high tidal volume and a longer stay on the ventilator were associated with a progress of lung injury. Moreover, the magnitude of COVID-19-associated lung injury correlated with several clinical features describing patients’ ability to oxygenate, ventilate and regulate fluid balance. Correlations between lung injury, inflammatory and thrombotic markers have been observed.

### Regional distribution of lung hypodensities

COVID-19-associated lung injury showed a specific sub-pleural distribution (Fig. 2 and Additional file 2: Figure S1) coexisting with a varying involvement of the core areas of the lung. This confirmed what has been previously described in a qualitative manner [7, 8, 20]. However, our results did not support a strict division between two respiratory phenotypes [7], but rather a more gradual evolution from a subpleural to a more central distribution of lung injury [8]. The prominent subpleural distribution was confirmed for both dependent and non-dependent lung regions and so unrelated to a gravitational distribution, which means we could exclude confounding by atelectasis and pleural effusion that typically affect the dependent regions of the lung. As already known, normoinflation was mainly distributed in subpleural regions [28]. To date, the reasons for a primary distribution of COVID-19-associated lung injury on subpleural regions are not fully clarified. Gattinoni and coworkers [7] speculated about the possibility that subpleural regions are areas of interface between lung structures with different elastic proprieties, hence converging higher stress and strain [29]. A complementary explanation is provided by the physics of the deposition of inhaled viral particles. Particles of the size of the virion (0.15 μm), deposit mainly in the terminal alveolar region [30], being scarcely affected by inertial impaction or gravitational sedimentation on airways walls. The following phase is characterized by an inflammatory pattern that spreads centripetally, possibly following the direction imposed by the ciliar beats on mucus and the watery periciliary liquid [31]. The higher proportion of injured central regions of the lung after 14 days of spontaneous breathing has been demonstrated in our study (Fig. 3).

### Correlations between lung compartments and clinical features

#### Dichotomous variables

Potentially dangerous ventilatory conditions seemed to favor the involvement of the core areas of the lung. The exposure to invasive mechanical ventilation per se, independently from its duration, was associated with an increased amount of injured lung (Fig. 3). Hypothesizing a causal association between these variables, one can speculate that the exposure to invasive ventilation can favor the progress of lung injury in COVID-19-associated acute respiratory failure as previously shown in other forms of ARDS [32]. However, an alternative explanation may be that patients with more advanced lung disease are more likely to need invasive ventilatory support compared to patients with a milder lung disease. For those patients undergoing invasive ventilation, we found that a less protective tidal volume [33], higher than 6 mL/kg PBW, was linked to an extended lung injury visible by CT (Fig. 3).

A prolonged time of spontaneous breathing before CT was another important condition related to the progression of lung injury (Fig. 3). A longer period of spontaneous breathing (more than 14 days) between the onset of the respiratory symptoms and the CT scan in patients in need of intensive care was associated with a progression of lung injury. Patients spontaneously breathing for less than 14 days may include patients intubated early, thereby having longer controlled ventilation as well as patients in an earlier stage of COVID-19. Therefore, injurious consequences of a prolonged uncontrolled spontaneous breathing ventilation for those patients that after 14 days of acute respiratory failure are still in need of intensive care can be hypothesized. The hyperpnoea, characterizing the COVID-19-associated acute respiratory failure, can be associated with an increased and dangerous transpulmonary pressure [34]. This prolonged high-stress ventilation, combined with the fluid leak characterizing the inflammatory state to which the lung is exposed, can in the end lead to P-SILI [32, 35].

The subgroup of patients showing high ferritin levels (higher than 1000 µg/L) the day of the CT scan was associated with a significantly larger lung injury (Fig. 3). Plasma ferritin is an important marker for several inflammatory diseases [36], and high ferritin levels have been demonstrated to be linked to the severity of COVID-19 disease [37].

### Continuous variables

A direct correlation between the extension of injured areas and the exposure to mechanical ventilation or to a non-protective tidal volume (Additional file 2: Tables S4 and S5) further corroborated the analysis based on dichotomous variables (Fig. 3). Our data suggested that protective ventilation with limited tidal volumes is of particular importance in COVID-19-associated acute respiratory failure. More liberal mechanical ventilator settings have been proposed for COVID-19-associated acute respiratory failure [9] although not supported by univocal evidence and in contrast with the necessity of keeping a tidal volume equal or lower than 6 mL/kg PBW [32, 38]. Our results do not support this more liberal ventilatory approach. Recent studies indicate the importance of a protective ventilation in ARDS patients [14, 39], including COVID-19 patients [14]. Even a short exposure to high intensity mechanical ventilation can potentially be harmful and associated with increased mortality, independently from the severity of the initial lung injury [14, 39].

We found a direct correlation between injured lung and patients’ pronation or muscle relaxation (Additional file 2: Table S4). This can be easily explained by the fact that more severe forms of SARS-CoV2 related acute respiratory failure are more likely in need of pronation [40] and more often undergo muscle relaxation as standard of care [41]. Nine (39%) out of 23 patients included in the study underwent pronation with improved oxygenation during their intensive care stay.

The direct correlation found between the onset of lung injury and a cumulative fluid balance (and need of RRT) confirmed the importance of avoiding fluid overload in patients with COVID-19-associated acute respiratory failure. The need for RRT is an indicator for acute kidney injury and most likely for a positive fluid balance. Previous investigations have shown that a positive fluid balance is associated with the increased risk of death, more days of mechanical ventilation and a longer ICU-stay in patients with ARDS [42, 43].

We did not find any correlation between the amount of lung injury and the tested inflammatory biomarkers (list in Additional file 2: Table S4). This supported previous results [44] suggesting that the severity of COVID-19-associated acute respiratory failure is poorly related to commonly used inflammatory biomarkers. Nevertheless, as stated before, the subgroup of patients showing ferritin levels higher than 1000 µg/L the day of the CT scan was associated with a significantly larger lung injury (Fig. 3).

Although not able to demonstrate a direct link between plasma D-dimers and thrombotic burden, we found that high plasma D-dimers levels correlated with lung injury detected by CT. A recent study by Grasselli and coworkers [14] shows that high plasma D-dimers are correlated to high 28-day mortality in COVID-19-associated acute respiratory failure. A number of changes toward a pro-thrombotic state, including an increased level of D-dimers and activation of the lectin pathway of complement activation, have been observed in patients with severe COVID-19 [45, 46]. The correlation found between lung injury and D-dimers was consistent with a spread phenomenon of pulmonary micro-thrombosis observed in histological studies [47]. Nonetheless, the activation of a pro-thrombotic state at the alveolar level is one of the events characterizing all forms of ARDS and leading to the intra-alveolar fibrin deposition, vascular injury and micro-thrombi occurrence [48].

The present study has different limitations. The subgroup of patients selected for the radiological investigations was sicker compared to the whole cohort, showing higher 20 day mortality (36% vs 16%) and SAPS3 (57 vs 51) (Table 1). Positive end-expiratory pressure levels and other ventilatory settings selected during the acquisition of chest CT scans varied depending on best clinical practice, adding an element of heterogeneity in the comparisons of different CT scans (Table 2). The CT scans were performed on clinical indication. The consequent non-standardized approach derived from the good clinical practice guidelines in force during the first pandemic wave when local and international routines for management and transportation of critically ill COVID-19 patients were still under development. Patients numerosity as well as the number of chest CT scans usable for the current analysis was limited by the necessity of including only chest CT scans acquired without contrast agent. The contrast mean can variably alter the HU scale depending on its blood concentration and the regional distribution of tissue. The inclusion of images containing contrast would have introduced a further source of heterogeneity whose resolution would have required rely upon assumptions and analysis whose complexity is beyond the scopes of the present contribution. The lack of a control group with classical ARDS made the comparison between COVID-19-associated acute respiratory failure and other forms of ARDS impossible. The correlations tested in this study did not necessarily imply causality. The choice of 14 days for estimating the influence of timing of mechanical ventilation is based on the evidence that in different studies this is the time point within which the most of the patients are intubated [27, 49].

## Conclusions

The current study quantified COVID-19-associated lung injury and emphasized the importance of chest CT in the clinical management of COVID-19 in intensive care. In a cohort of COVID-19 patients with severe respiratory failure, a predominant subpleural distribution of lung injury was observed, associated with a variable involvement of more central regions. COVID-19-associated lung injury correlated with: 1) exposure to mechanical ventilation, 2) ventilatory patterns potentially leading to P-SILI, 3) pro-thrombotic conditions. The practical implications of the present study are: 1) chest CT is useful to infer the course of the disease; 2) long duration of spontaneous breathing and high tidal volumes in critically ill COVID-19 patients may increase the spread of lung alterations centripetally.

## Supplementary Information

**Additional file 1.** For additional information about Materials and methods

**Additional file 2.** For additional tables and figures

## Data Availability

The datasets used and/or analyzed during the current study are available from the corresponding author on reasonable request.

## Abbreviations

ARDS: Acute respiratory distress syndrome
COVID-19: Coronavirus disease 2019
CT: Computed tomography
EPM: Ethical review agency
HU: Hounsfield units
ICU: Intensive care units
IQR: Interquartile ranges
P-SILI: Patient self-induced lung injury
PBW: Predicted body weight
PCR: Polymerase chain reaction
ROI: Region of interest
RRT: Renal replacement therapy
R_s_: Pairwise linear correlation coefficients
SAPS3: Simplified acute physiology score 3
SARS-CoV2: Severe acute respiratory syndrome coronavirus 2
