# Supplementary material for: A quantitative analysis of extension and distribution of lung injury in COVID-19: a prospective study based on chest computed tomography
Source: Crit Care. 2021 Aug 4;25:276. doi: 10.1186/s13054-021-03685-4 (PMC8334337; doi:10.1186/s13054-021-03685-4)
Supplement: Supplementary file 1 — Additional file 1. For additional information about Materials and methods [file 13054_2021_3685_MOESM1_ESM.docx]

**A quantitative analysis of extension and distribution of lung injury in COVID-19: a prospective study based on chest computed tomography.**

**- Additional file 1 -**

Mariangela Pellegrini, MD, PhD; Aleksandra Larina, MD; Evangelos Mourtos, MD; Robert Frithiof, MD, PhD; Miklos Lipcsey, MD, PhD; Michael Hultström, MD, PhD; Monica Segelsjö; Tomas Hansen, MD, PhD; Gaetano Perchiazzi MD, PhD;

**Materials and methods**

This study was performed in patients admitted to the intensive care units (ICU) of Uppsala University Hospital (Anesthesia, Operation and Intensive Care Department, Akademiska Sjukhuset, Uppsala, Sweden). The study was approved by the National Ethical Review Agency (EPM; No. 2020-01623). The Declaration of Helsinki and its subsequent revisions were followed. The protocol was registered *a priori* (Clinical Trials ID: NCT04316884). STROBE guidelines were followed for reporting. Written informed consent was obtained from the patients when possible. Otherwise, informed consent was firstly asked to next to kin and later confirmed by patients if feasible. Selected patients were older than 18 years, positive PCR test for SARS-CoV2 on nasal swab specimen, admitted to intensive care unit between March 13^th^ and June 5^th^, 2020 and undergone a spiral chest CT (Somatom Definition Flash, Siemens AG, Erlangen, Germany). The CT scans were performed without contrast agent, covering the whole lung parenchyma and acquired in supine position. CT parameters were: 120 kV, CTDI vol 2.87, image recon i70f 4, axial images 3 mm slice thickness. Spiral CT scans were performed when clinically indicated and technically feasible, hence at different timing and different stages of the disease.

Laboratory, clinical and physiological variables

Comprehensive clinical data about the included patients (e.g. demographics, chronic health conditions, vital signs, ventilatory settings and laboratory tests, including markers of acute inflammation) were collected on daily basis. The list of the acquired clinical data was reported in *Tables 1 and 2*.

CT image analysis

The sequences of images obtained during the spiral rotation of the CT detector were stored in DICOM format and processed as two-dimensional matrices in MatLab environment (MatLab and Statistics Toolbox Release 2020a, The MathWorks, Natick, USA). The collected CT images were two-dimensional square matrices (512 x 512 voxels) having as voxel dimensions 0.7461 mm x 0.7461 mm x 1 mm. Twenty images evenly spaced along the cranial-caudal axis and located between the apex (as cranial limit) and the diaphragmatic dome (as caudal limit) were selected for analysis. We deemed twenty levels to be qualitatively representative of the entire lung according to an interpolation principle already applied by Reske et al. [1] and later verified by Ball et al. [2]. A manual lung parenchyma segmentation was applied, and big vessels, heart and mediastinal structures were excluded from the analysis.

In order to describe the distribution of lung lesions along the sub pleural-to-core direction inside lung parenchyma, three concentric subpleural regions of interest were defined: from pleural surface to 1 cm depth; from 1 to 2 cm depth; and from 2 to 3 cm depth, parallel to the pleural surface (*Figure1*). To distinguish between lung injury and dependent atelectasis and pleural effusion, each lung depicted in CT images was also divided into four quadrants. The quadrants were defined by lines passing through the centroid of the lung (also defined as geometrical center) univocally characterizing each single lung image. The quadrants identified by this method were labeled as internal dependent, external dependent, external non-dependent, internal non-dependent (*Figure1*). Consequently, three functional regions were identified by grouping these quadrants: *dependent* (internal dependent + external dependent), *non-dependent* (external non-dependent + internal non-dependent) and *external* (external non-dependent + external dependent). Concentric ROIs, quadrants and lung regions were therefore combined to make possible a regional analysis of the distribution of lung injury (*Figure1*).

Lung compartments

Each voxel composing the CT scans was characterized by a CT number expressed as Hounsfield units (HU). Following a previous convention [3], four lung compartments were defined: hyperinflated (HU between –1,000 and –800), normoinflated (HU between –800 and –500), hypoinflated (HU between –500 and –100) and not inflated or atelectasis (HU between –100 and +100) [4]. The extension of each lung compartment was expressed as percentage [%] of the total lung volume analyzed in a given chest CT slice (defined in figures 2 and 3 as *% of total lung volume*).

For each of the 20 slices selected per CT scan, lung volume, gas volume and lung weight have been calculated according to established methods [1,2,5] applying the following equations:

Volume of gas = voxel volume * voxel attenuation /-1000

Tissue weight = voxel volume * (1 - (voxel attenuation /-1000))

When the voxel volume is expressed as cubic centimeters [cm^3^], the volume of gas is in milliliters [ml] and the tissue weight is in grams [g]; voxel attenuation is expressed as Hounsfield Units [HU].

Applying the above mentioned equations, total lung volume, total gas volume and total lung weight have been then calculated from the sequence of CT scans, following the interpolation method reported by Ball et al [2] and by Reske et al [1]:

$$Mlung= \sum_{i=1}^{N-1} \left( f* \frac{M_{i}+M_{i+1}}{2*t} \right)+ \frac{M_{1}+M_{N}}{2}$$

Where *N* is the number of slices, *t* is the slice thickness, *f* the distance between slices and *Mi* the lung mass in the *i^th^* slice. This equation yields the total mass of the lung *M_lung_*. This general interpolation method is also able to compute total lung volume and total gas volume, when the mass *Mi* is appropriately substituted by respectively the lung volume or the gas volume in the *i^th^* slice.

The mentioned equation was used for computing lung volume, gas volume and lung weight for the entire lung as well as for each lung aeration compartment. The latter have been reported both as absolute values and as percentage of the total lung parenchyma. The extension of pathologically altered lung was defined as the sum of hypoinflated and not inflated (atelectatic) parenchyma distributed in not-dependent or external regions in accordance to the principles expressed by Gattinoni [6] and Rouby [7]. Moreover, the not inflated lung compartment was analyzed for both the whole studied population and for patients’ subgroups. Subgroups were defined based on dichotomous variables: 1) exposure to mechanical ventilation before CT scan (yes/not); 2) exposure to a mean tidal volume higher/lower than 6 mL/kg predicted body weight (PBW); 3) duration of spontaneous breathing between the onset of symptom reported by the patient and the CT scan for more/less than 14 days; 4) plasma level of ferritin lower/higher than 1,000 µg/L (normal plasma ferritin range: 12 to 150 µg/L). Spontaneous breathing, before invasive ventilation, was defined as all types of assisted and not-assisted non-invasive ventilation. The selected thresholds for spontaneous breathing duration and for plasma ferritin were based on previous literature [8].

Statistical analysis

Data analysis and statistical tests were performed using dedicated MatLab scripts (MatLab and Statistics Toolbox Release 2020a, The MathWorks, Natick, USA). Continuous variables were reported as medians and interquartile ranges (IQR) and represented as boxplots. All statistical tests were two sided; p < 0.05 was considered statistically significant. A two-way analysis of variance for not parametric data (Friedman's test) was used to detect statistical differences in HU distribution among the three subpleural layers and the whole lung parenchyma. Subsequently, pairwise comparisons, based on the Bonferroni method, were executed when analysis of variance detected a significant difference inside the tested group of ROIs. A Wilcoxon rank sum non-parametric test was used to evaluate statistical differences between subgroups defined by dichotomous variables. Spearman correlation test was used to analyze correlations between lung compartments and selected clinical data. The list of clinical data included in the analysis was reported in *supplementary table 3*. Non-parametric correlation was the test of choice because at least one of the compared variables was not normally distributed. Pairwise linear correlation coefficients (R_s_) and p-values were collected and interpreted based on value α= 0.05.

**References**

1. Reske A, Reske A, Gast H, Seiwerts M, Beda A, Gottschaldt U, et al. Extrapolation from ten sections can make CT-based quantification of lung aeration more practicable. Intensive Care Medicine. 2010;36:1836–44.

2. Ball L, Braune A, Corradi F, Brusasco C, Garlaschi A, Kiss T, et al. Ultra-low-dose sequential computed tomography for quantitative lung aeration assessment—a translational study. Intensive Care Medicine Experimental. 2017;5:19.

3. Gattinoni L, Caironi P, Pelosi P, Goodman LR. State of the Art What Has Computed Tomography Taught Us about the Acute Respiratory Distress Syndrome ? 2001;164:1701–11.

4. Gattinoni L, Pesenti A, Bombino M, Baglioni S, Rivolta M, Rossi F, et al. Relationships between lung computed tomographic density, gas exchange, and PEEP in acute respiratory failure. Anesthesiology. 1988;69:824–32.

5. Rylander C, Högman M, Perchiazzi G, Magnusson a, Hedenstierna G. Oleic acid lung injury: a morphometric analysis using computed tomography. Acta anaesthesiologica Scandinavica [Internet]. 2004 [cited 2014 Sep 24];48:1123–9. Available from: http://www.ncbi.nlm.nih.gov/pubmed/15352958

6. Gattinoni L, Presenti A, Torresin A, Baglioni S, Rivolta M, Rossi F, et al. Adult respiratory distress syndrome profiles by computed tomography. Journal of Thoracic Imaging. 1986;1:25–30.

7. Rouby J-J, Puybasset L, Nieszkowska A, Lu Q. Acute respiratory distress syndrome: Lessons from computed tomography of the whole lung. Critical Care Medicine. 2003;31:S285–95.

8. Zhou F, Yu T, Du R, Fan G, Liu Y, Liu Z, et al. Clinical course and risk factors for mortality of adult inpatients with COVID-19 in Wuhan, China: a retrospective cohort study. www.thelancet.com. 2020;395.

**Supplementary Figure caption**

**Supplementary Figure E1.** **Boxplot showing the regional distribution of lung compartments (hyper, normo, hypo and non-inflated lung) in A) whole lung; B) external regions of the lung.** Moreover, each subgroup of boxplot shows how lung compartments are divided among different subpleural regions of interest (blue for 0-1 cm subpleural area, green for 1-2 cm subpleural area, orange for 2-3 cm subpleural area, black for the whole analyzed area). The regional distribution of lung compartments is expressed in % of total lung volume (y-axis) for all the reported graphs. Friedman's test was used to detect statistical differences. Pairwise comparisons were performed if analysis of variance detected a significant difference inside the tested group of ROIs. Adjustment for multiple comparisons was applied according to the Bonferroni method. *: to indicate statistical difference.

**Supplementary Figure E2.** **Boxplot showing the regional distribution of lung compartments (hyper, normo, hypo and non-inflated lung) in A) non-dependent regions; B) dependent regions of the lung.** For detailed information, refer to Figure E1.

**Supplementary Tables captions**

**Supplementary Table E1.** **HU distribution among the three subpleural layers and the whole lung parenchyma.** A two-way analysis of variance for not parametric data (Friedman's test) was used. Pairwise comparisons were, then, used if analysis of variance detected a significant difference inside the tested group of ROIs. p-values are reported in the tables.

**Supplementary Table E2. Differences between dependent and non-dependent lung regions, analyzed as whole parenchyma and as divided into concentric subpleural regions of interest (ROIs).** The Wilcoxon rank sum non-parametric test (α=0.05) was used to evaluate statistical differences. p-values are reported. For graphical visualization see *Figure E2*.

**Supplementary Table E3.** **Statistical differences in not-inflated lung compartment between subgroups defined by dichotomous variables.** The Wilcoxon rank sum non-parametric test was used. Statistical differences have been checked for different distances from the pleural and for the analyzed lung region. p-values are reported in the tables. For graphical visualization see *Figure 3*.

**Supplementary Table E4.** **List of variables tested for correlation with lung compartments.** The Spearman correlation test was applied to obtain pairwise linear correlation coefficients (Rs) and p-values to define statistical significance based on α-value < 0.05. PEEP: positive end-expiratory pressure; PWB: predicted body weight; PCO_2_: partial pressure of carbon dioxide; SpO_2_: peripheral oxygen saturation.

**Supplementary Table E5.** **Statistically significant correlations between lung compartments (hyper, normo, hypo and not inflated) and clinical features, tested in the whole lung parenchyma.** The Spearman correlation test (α-value < 0.05) was used to test statistical significance. Rs: pairwise linear correlation coefficients; CT: computed tomography; PWB: predicted body weight; PCO_2_: partial pressure of carbon dioxide; SpO_2_: peripheral oxygen saturation.
