# Supplementary material for: A quantitative analysis of extension and distribution of lung injury in COVID-19: a prospective study based on chest computed tomography
Source: Crit Care. 2021 Aug 4;25:276. doi: 10.1186/s13054-021-03685-4 (PMC8334337; doi:10.1186/s13054-021-03685-4)
Supplement: Supplementary file 2 — Additional file 2. For additional tables and figures [file 13054_2021_3685_MOESM2_ESM.pptx]

## Slide 1
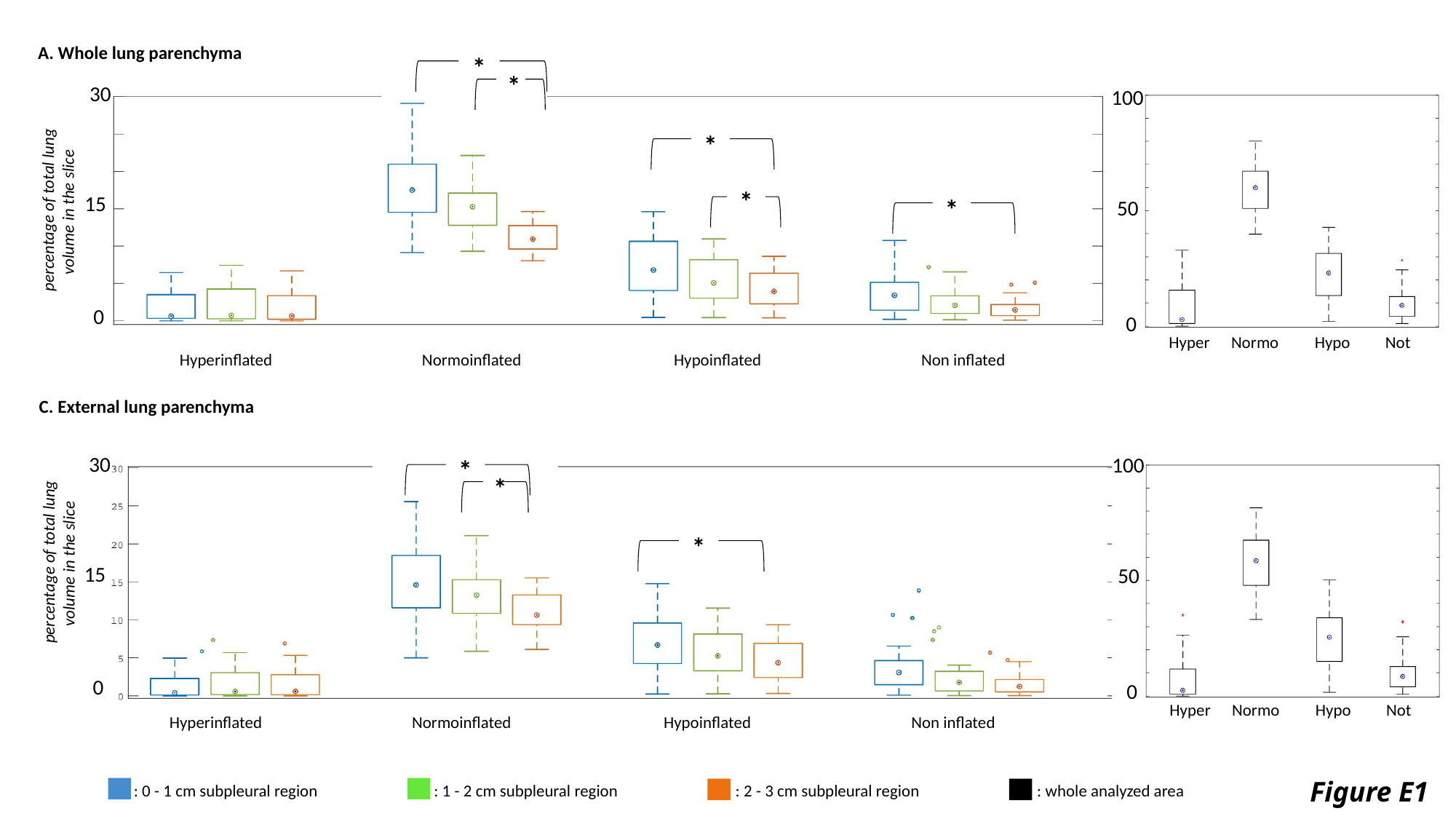

A. Whole lung parenchyma
*
*
*
30
15
0
100
50
0
*
*
percentage of total lung volume in the slice
*
Hyper
Normo
Hypo
Not
Hyperinflated
Normoinflated
Hypoinflated
Non inflated
C. External lung parenchyma
30
15
0
100
50
0
*
*
*
percentage of total lung volume in the slice
Hyper
Normo
Hypo
Not
Hyperinflated
Normoinflated
Hypoinflated
Non inflated
Figure E1
: 0 - 1 cm subpleural region
: 1 - 2 cm subpleural region
: 2 - 3 cm subpleural region
: whole analyzed area

## Slide 2
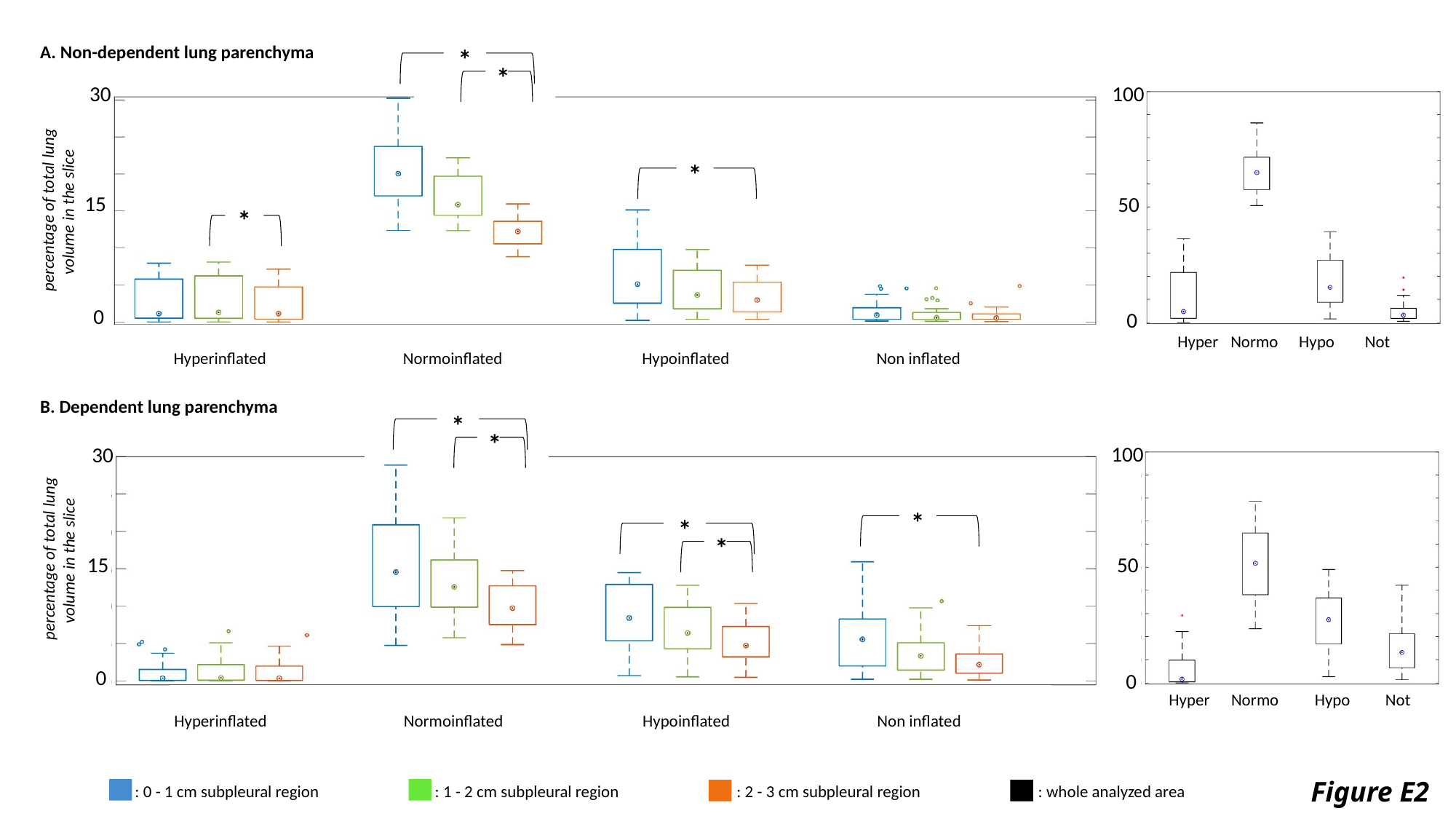

A. Non-dependent lung parenchyma
*
*
30
15
0
100
50
0
*
percentage of total lung volume in the slice
*
Hyper
Normo
Hypo
Not
Hyperinflated
Normoinflated
Hypoinflated
Non inflated
B. Dependent lung parenchyma
*
*
100
50
0
30
15
0
*
*
*
percentage of total lung volume in the slice
Hyper
Normo
Hypo
Not
Hyperinflated
Normoinflated
Hypoinflated
Non inflated
Figure E2
: 0 - 1 cm subpleural region
: 1 - 2 cm subpleural region
: 2 - 3 cm subpleural region
: whole analyzed area

## Slide 3
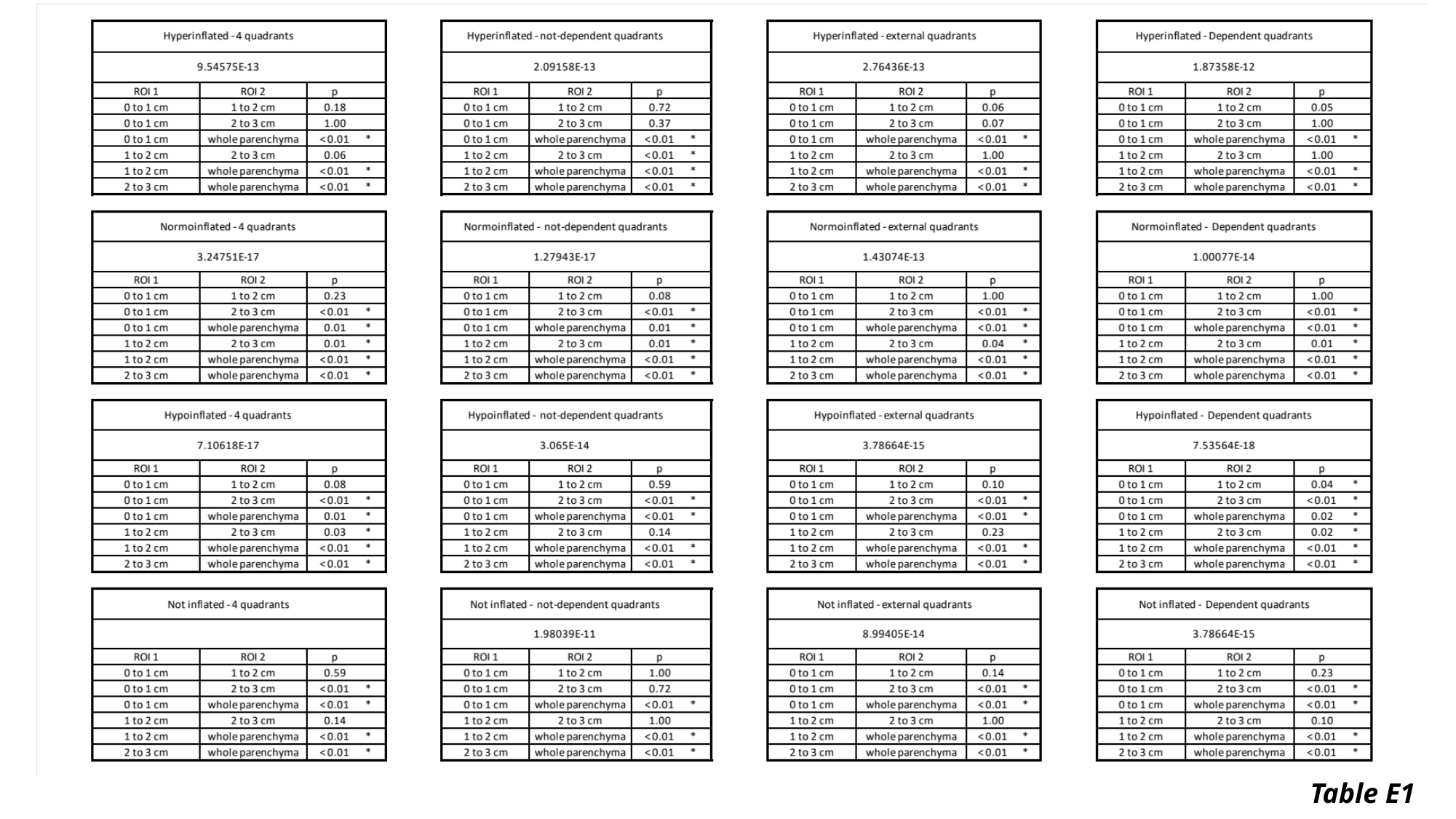

Table E1

## Slide 4
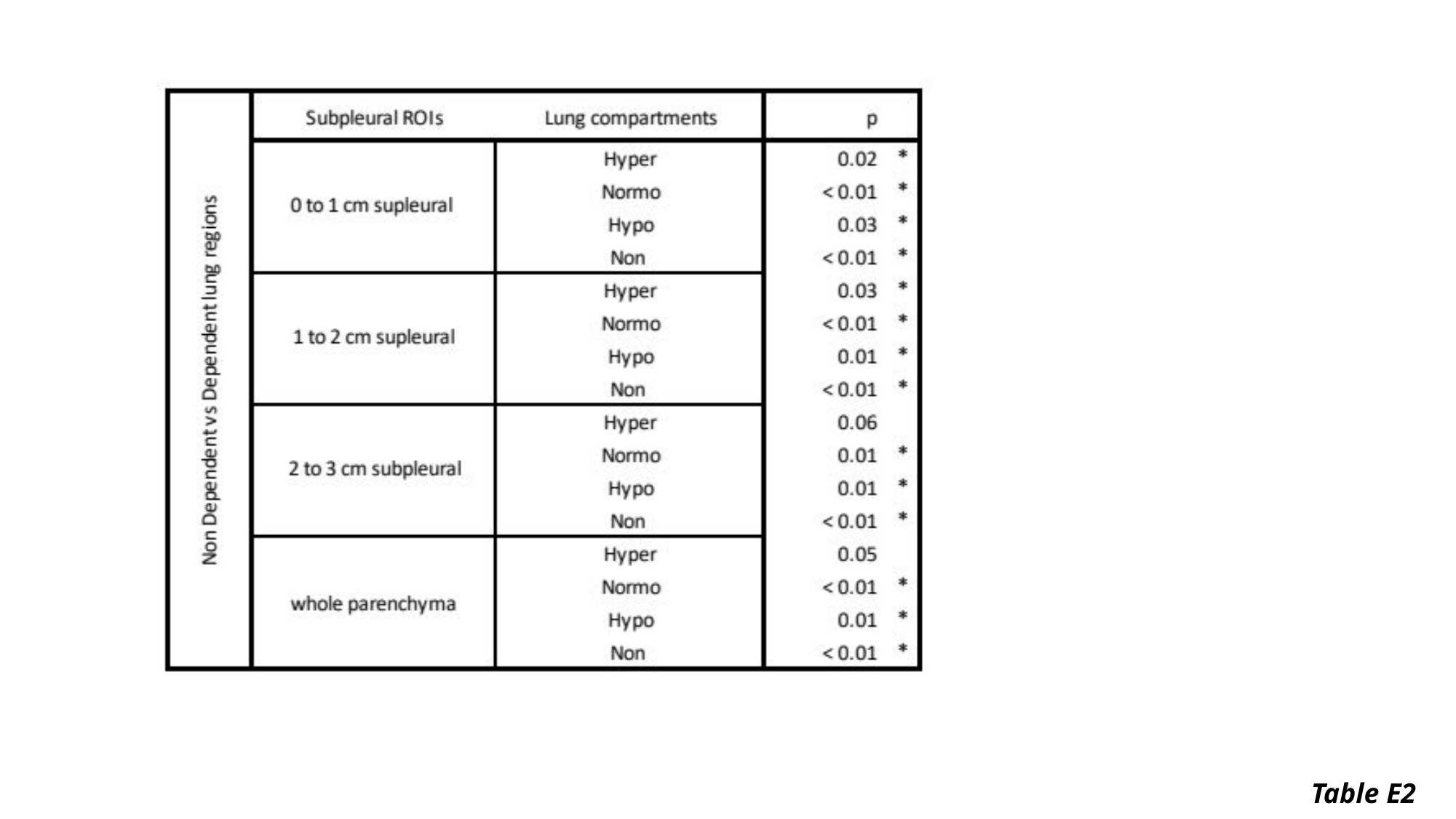

Table E2

## Slide 5
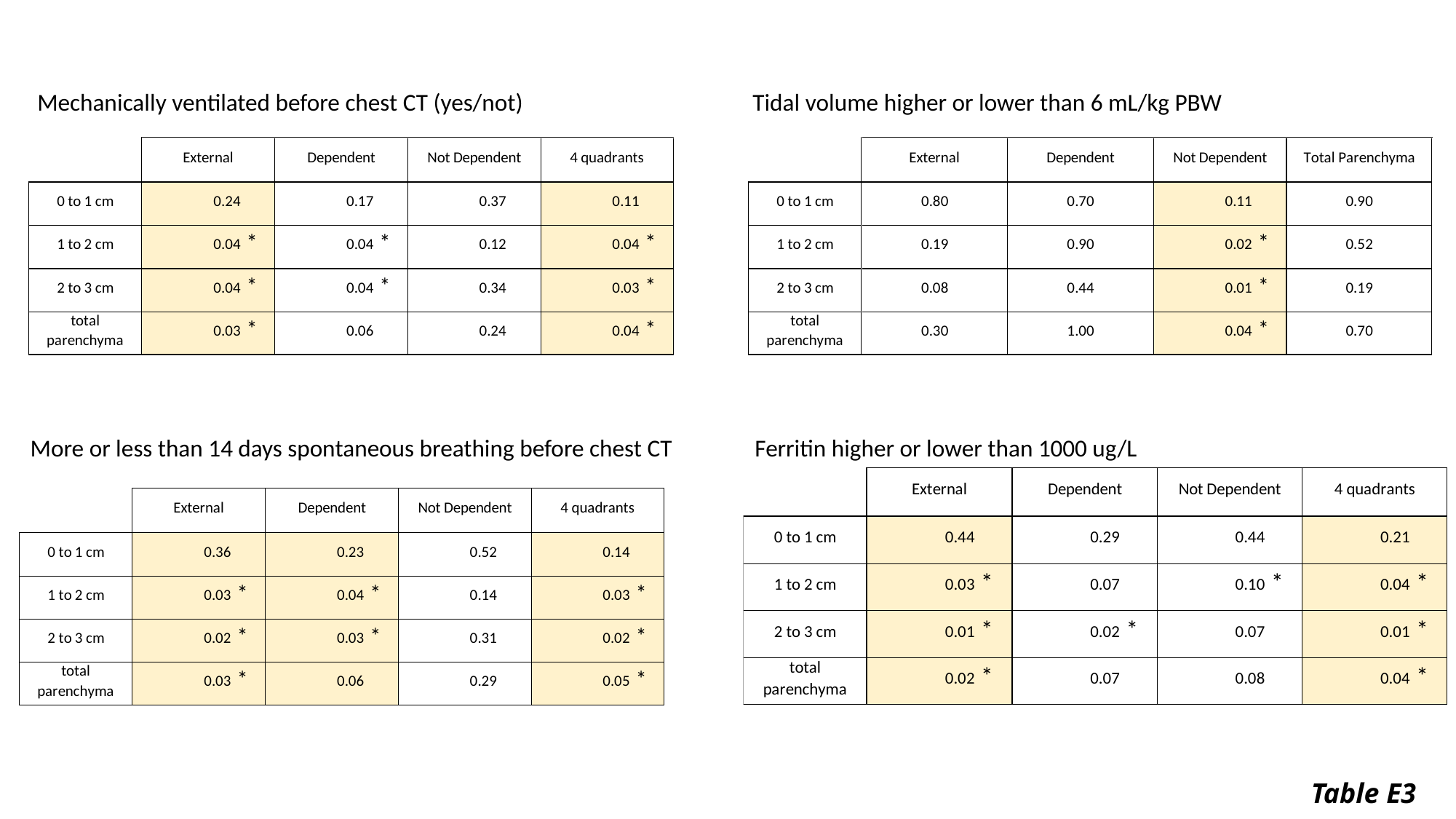

Mechanically ventilated before chest CT (yes/not)
Tidal volume higher or lower than 6 mL/kg PBW
More or less than 14 days spontaneous breathing before chest CT
Ferritin higher or lower than 1000 ug/L
Table E3

## Slide 6
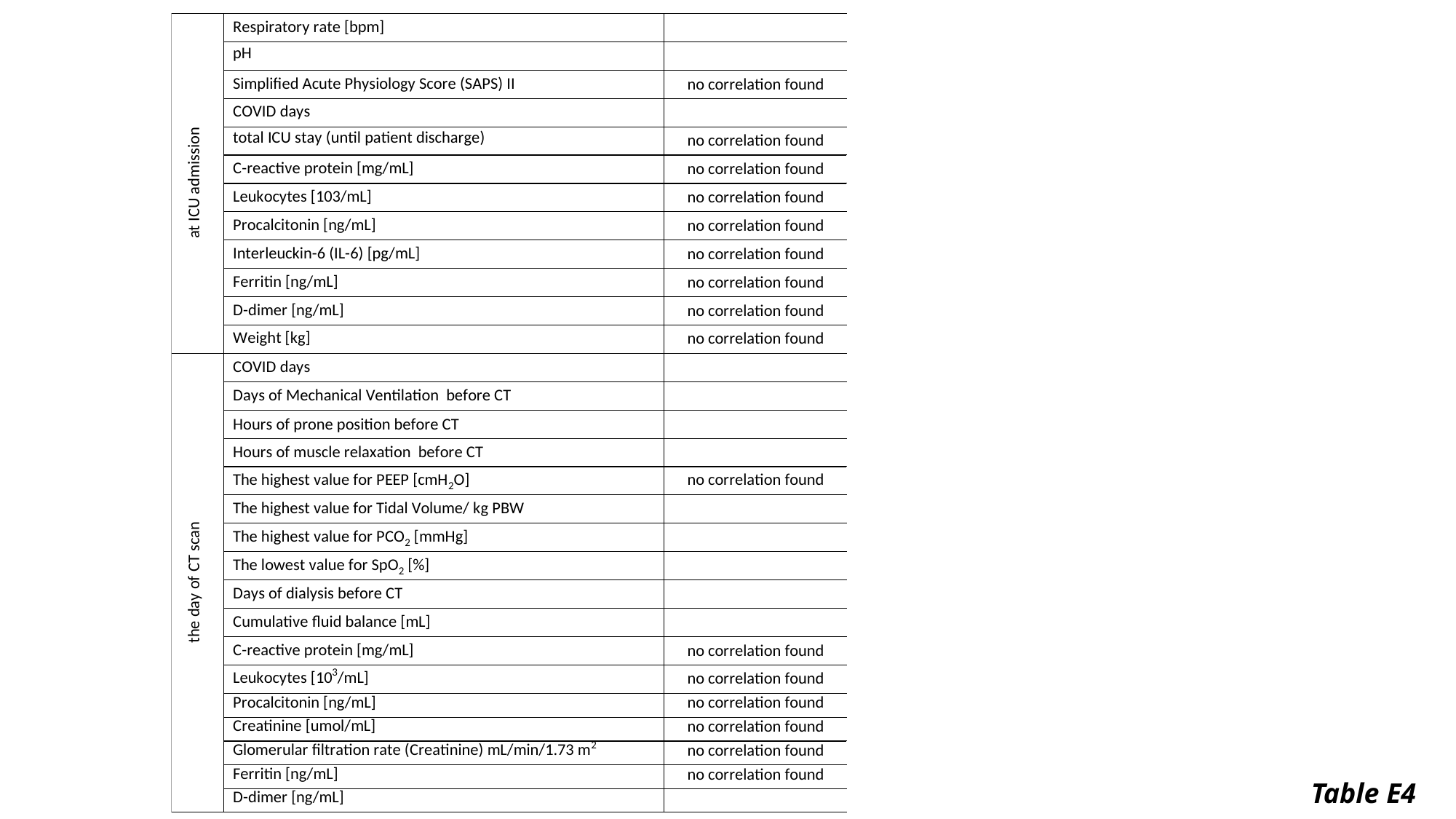

Table E4

## Slide 7
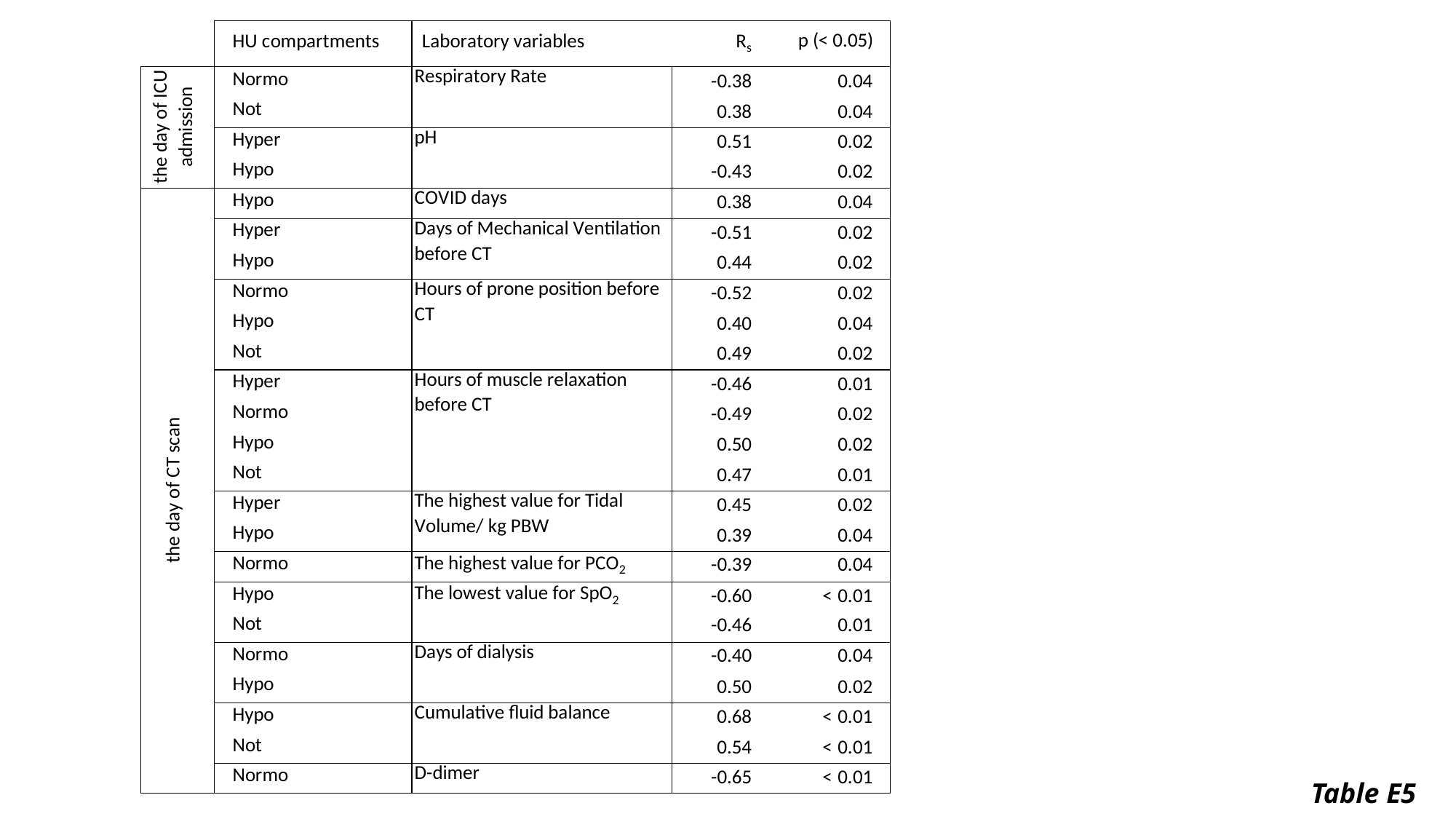

Table E5
